# Extending schizophrenia diagnostic model to predict schizotypy in first-degree relatives

**DOI:** 10.1038/s41537-020-00119-y

**Published:** 2020-11-06

**Authors:** Sunil Vasu Kalmady, Animesh Kumar Paul, Russell Greiner, Rimjhim Agrawal, Anekal C. Amaresha, Venkataram Shivakumar, Janardhanan C. Narayanaswamy, Andrew J. Greenshaw, Serdar M. Dursun, Ganesan Venkatasubramanian

**Affiliations:** 1 Alberta Machine Intelligence Institute, University of Alberta, Edmonton, AB Canada; 2 Canadian VIGOUR Centre, University of Alberta, Edmonton, AB Canada; 3 Department of Computing Science, University of Alberta, Edmonton, AB Canada; 4 Department of Psychiatry, University of Alberta, Edmonton, AB Canada; 5 Translational Psychiatry Laboratory, Neurobiology Research Centre, National Institute of Mental Health and Neuro Sciences, Bangalore, India; 6 Department of Sociology and Social Work, Christ- Deemed to be University Bangalore, Bangalore, India; 7 Schizophrenia Clinic, Department of Psychiatry, National Institute of Mental Health and Neuro Sciences, Bangalore, India; 8 Department of Integrative Medicine, National Institute of Mental Health and Neuro Sciences, Bangalore, India

**Keywords:** Biomarkers, Human behaviour, Schizophrenia

## Abstract

Recently, we developed a machine-learning algorithm “EMPaSchiz” that learns, from a training set of schizophrenia patients and healthy individuals, a model that predicts if a novel individual has schizophrenia, based on features extracted from his/her resting-state functional magnetic resonance imaging. In this study, we apply this learned model to first-degree relatives of schizophrenia patients, who were found to not have active psychosis or schizophrenia. We observe that the participants that this model classified as schizophrenia patients had significantly higher “schizotypal personality scores” than those who were not. Further, the “EMPaSchiz probability score” for schizophrenia status was significantly correlated with schizotypal personality score. This demonstrates the potential of machine-learned diagnostic models to predict state-independent vulnerability, even when symptoms do not meet the full criteria for clinical diagnosis.

## Introduction

Genetic inheritance plays a strong role in the etiology of schizophrenia, representing ~80% of the liability for the illness, based on numerous twin and adoption studies^1–3^. Recent studies demonstrated that first-degree relatives of schizophrenia patients are more likely to exhibit associated intermediate phenotypes or “endophenotypes”, than the general population, even when they do not (or do not yet) present with a full set of clinical symptoms^4^. Numerous endophenotypes have been proposed in schizophrenia, including brain structural or functional patterns, sensory processing measures, neuromotor and neuropsychological measures, minor physical anomalies^5,6^. As such endophenotypic signatures can enable prediction systems that are neurobiologically consistent, it is important to investigate how such populations would be classified by a machine-learned model that is capable of distinguishing schizophrenia from healthy controls based on resting-brain activation patterns. Moreover, such explorations can shed light on the role of machine-learning models in identifying clusters of personality traits or subclinical symptoms in the general population. Motivated by this idea, this study examines whether a schizophrenia diagnosis model, learned using schizophrenia and normal functional magnetic resonance imaging (MRI) data sets, can identify higher schizotypal scores in first-degree relatives without schizophrenia.

Schizophrenia spectrum disorders (SSD) present a challenge in categorizing disease phenotypes, owing to a wide range of overlapping symptoms and the heterogeneous illness course at the individual level. The origin, development, and heterogeneity of SSD can be understood through the concept of “schizotypy”^7^, which is a multidimensional construct that encompasses several facets of personality organization, spanning from healthy variation to psychotic disorder^8^. Understanding the components of schizotypy holds great potential for early diagnosis and understanding of disease processes in SSD^9^. In recent years, several studies have examined neural correlates of schizotypy using resting-state fMRI (Supplementary Table 1). Further, there is increased interest in learning models from functional neuroimaging to predict schizotypy–with unfortunately limited generalizability owing to small training samples and lack of independent validation (for review, see ref. ^9^).

The current study explores an alternative approach for predicting the degree of schizotypal expression in unaffected first-degree relatives of schizophrenia patients. We applied the machine-learned diagnostic model that was trained on an independent resting-state fMRI data set of 81 antipsychotic-naive schizophrenia patients and 93 healthy controls. Given the strong evidence for familial aggregation of higher schizotypy expression in SSD^10^, we hypothesize that the first-degree relatives who were predicted by the model to have “schizophrenia” status, i.e., false positives (FP) will have significantly higher schizotypal scores, versus those who are predicted as non-schizophrenia status, i.e., true negatives (TN) by machine learning.

**Fig. 1:**
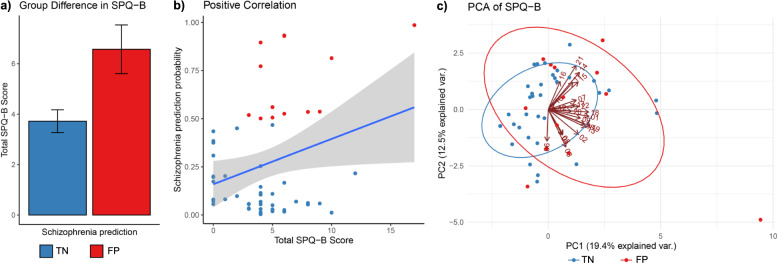
Relationship between schizophrenia prediction and SPQ-B score. **a** Bar graph shows significant mean difference in SPQ-B between predicted groups (error bars indicate standard error of mean); **b** scatter plot shows positive correlation between schizophrenia prediction probability and SPQ-B (gray band indicate 95% confidence interval); **c** PCA biplot with scores for study participants in predicted groups overlaid with loadings indicating influence of individual SPQ-B components on the principal components 1 and 2.

This model classified 14 out of 57 subjects as FP, whereas the remaining 43 were classified as TN, based on the default threshold level of schizophrenia prediction probability >0.5. We found that the FP group had a significantly higher total Schizotypal Personality Questionnaire—Brief (SPQ-B)^11^ score than that of TN (two-tailed *t* = 2.67, *p* = 0.01, Fig. 1a); similarly, there was a significant positive correlation between the probability of schizophrenia class and total SPQ-B score (Pearson’s *r* = 0.28, *p* = 0.03, Fig. 1b). FP and TN subjects did not differ significantly on age (two-tailed *t* = 1.02, *p* = 0.31) or sex distribution (*χ*^2^ = 0.32, *p* = 0.57).

To understand the effect of this machine classification further in relation to the latent structure of the SPQ-B questionnaire, we conducted a principal component analysis (PCA) of the 22 SPQ items. Figure 1c shows the biplot of two components of PCA (PC1 and PC2) along with the loadings of individual items as numbered arrows and the ellipses corresponding to FP or TN groups. In general, we observed that FP tended to show higher scores on PC1 comprising of items such as “unable to get close to people” (item-18), “people find me aloof and distant” (item-1), “often pick up hidden threats” (item-9) and “tend to keep my feelings to myself” (item-22); strikingly, these items relate more towards the negative schizotypy^12,13^. Review of studies on factors predicting the psychosis conversion suggests these items are amongst the significant predictors^14^. We have provided the full and summarized data on demographic, SPQ-B and the Structured Interview for Psychosis-risk Syndromes (SIPS)^15^ scores for the study participants (Supplementary Data 1 and 2, Supplementary Table 2) as well as some information on normative SPQ scores (Supplementary Table 3).

Schizotypy, especially the negative component^13^ is considered as a marker of vulnerability for schizophrenia that runs within families^10^. Furthermore, it provides a useful framework to investigate the etiological factors of SSD^16^. This study, for the first time, demonstrates a cross-application of a machine-learned schizophrenia diagnostic model in identifying subjects with high levels of negative schizotypy. However, whether similar prediction performance holds for a larger population without familial association remains to be explored. Further application of this approach holds significant promise for exploring related and comorbid symptom clusters in psychiatry.

## Methods

### Subjects

This study examined 57 first-degree relatives of schizophrenia patients (M:F = 42:15) based on the following inclusion and exclusion criteria. We included siblings or children of schizophrenia patients, without any axis-1 disorder as evaluated by the Mini International Neuropsychiatric Interview (MINI) Plus^17^. Probands of these participants were patients attending the clinical services of the National Institute of Mental Health & Neurosciences (NIMHANS), India, who fulfilled DSM-IV criteria for schizophrenia. The SIPS scale^15^ was administered to ascertain that these participants were unaffected by active psychosis. All except two subjects met criteria for “Genetic Risk and Deterioration Prodromal Syndrome”, while one subject met criteria for “Attenuated Positive Symptom Prodromal Syndrome” and another for “Brief Intermittent Psychotic Symptom Prodromal Syndrome”. We recruited only right-handed subjects to avoid potential confounds of differential handedness. No study subjects had contraindications to MRI or medical illness that could significantly influence brain structure/function, such as seizure disorder, cerebral palsy, or history suggestive of delayed developmental milestones. There was no history suggestive of DSM-IV psychoactive substance dependence or of head injury associated with loss of consciousness longer than 10 min. No participant had abnormal movements as assessed by the Abnormal Involuntary Movements Scale. Pregnant or postpartum females were not included. The age range was 17–38 years (27.2 ± 5.25 years). A 22-item self-reported screening measure of schizotypal personality traits—Schizotypal Personality Questionnaire—Brief (SPQ-B)^11^—was used to assess the schizotypal personality score as an estimator of schizotypal expression for each participant (range of total score: 0–22). The catchment area for the subject recruitment involved the southern states of India. We obtained informed written consent after providing a complete description of the study to all the subjects. The NIMHANS ethics committee reviewed and approved the original research protocol. The Research Ethics Board at the University of Alberta, Edmonton approved the secondary analysis of archived data.

### Image acquisition

MRI was done in a 3.0 Tesla scanner (Magnetom Skyra, Siemens). Resting-state fMRI: blood oxygen level dependent-sensitive echo-planar imaging was obtained using a 32-channel coil for a duration of 5 min 14 s, yielding 153 dynamic scans. The scan parameters were: TR = 2000 msec; TE = 30 msec; flip angle = 78 degrees; slice thickness = 3 mm; slice order: descending; slice number = 37; gap = 25%; matrix = 64 × 64 × 64 mm^3^, FOV = 192 × 192, voxel size = 3.0 mm isotropic. Subjects were asked to keep their eyes open during the scan. For intra-subject co-registration, structural MRI: T1 weighted three-dimensional high-resolution MRI was performed (TR = 8.1 msec, TE = 3.7 msec, nutation angle = 8 degree, FOV = 256 mm, slice thickness = 1 mm without inter-slice gap, NEX = 1, matrix = 256 × 256) yielding 165 sagittal slices.

### Image pre-processing

We visually inspected the acquired images for artifacts such as incomplete brain coverage or ghosting; then re-orientated the origin to the anterior commissure in structural MRI and fMRI images. Then, for each subject, we discarded the first 10 volumes of each functional time-series before reaching steady magnetization and for allowing participants to adapt to scanning noise. Images were then pre-processed with slice-timing correction and image realignment to correct for motion. Functional images were co-registered with the structural image and then normalized to MNI space resampled to 3 × 3 × 3 mm^3^. Further, we performed nuisance regression to denoise signal induced by head motion using 24 regressors derived from the parameters estimated during motion realignment, scanner drift using a linear term, as well as global fMRI signals from white matter and cerebrospinal fluid segments using SPM’s new segment method^18^. Finally, we smoothed, detrended and band-pass filtered (0.01–0.08 Hz) the normalized images. Software packages used for pre-processing and feature extraction are Statistical parametric mapping (SPM8, http://www.fil.ion.ucl.ac.uk/spm), Data Processing Assistant for Resting-State fMRI^19^, and nilearn python package^20^.

### Machine-learned prediction

We applied the learned EMPaSchiz model^21^ to classify each participant either as schizophrenia patient, i.e., FP or healthy individual, i.e., TN; and examined if there is a class difference, between the FP and TN individuals, in the distribution of SPQ-B^11^ scores. Note that none of the subjects in this study were in the training set used to produce the EMPaSchiz model (as a schizophrenia patient or healthy control).

### Reporting summary

Further information on research design is available in the Nature Research Reporting Summary linked to this article.

## Supplementary information

Supplementary Material

Reporting Summary

Supplementary Data 1

Supplementary Data 2

## Data Availability

The data sets generated during and/or analyzed during the current study are available from corresponding authors on a reasonable request.

## Code availability

Relevant computer codes that were used to process the data and to generate the results are available from corresponding authors on a reasonable request.
