# Supplementary material for: Extending schizophrenia diagnostic model to predict schizotypy in first-degree relatives
Source: NPJ Schizophr. 2020 Nov 6;6:30. doi: 10.1038/s41537-020-00119-y (PMC7648110; doi:10.1038/s41537-020-00119-y)
Supplement: Supplementary file 1 — Supplementary Material [file 41537_2020_119_MOESM1_ESM.pdf]

Supplementary Materials

Extending Schizophrenia diagnostic model to predict Schizotypy in first degree relatives

----

Supplementary Data 1: Complete data on demographic and Schizotypal Personality Questionnaire – Brief (SPQ-B) for the study participants.

Supplementary Data 2: Complete data on the Structured Interview for Psychosis-risk Syndromes (SIPS) scores for the study participants.

Supplementary Table 1: Recent studies examining schizotypy or schizotypal disorder with resting-state fMRI

Study	Year	Study groups	Implicated brain regions
Wang Y[1]	2020	12 schizotypal disorder in children and 9 typically developing children	Reduced grey matter volume in superior and medial frontal gyri<br><br>Increased resting-state functional connectivity between the superior frontal gyrus and inferior parietal lobule
Hua JPY[2]	2019	18 individuals with psychosis risk and 19 healthy individuals	Decreased striatal-cortical DMN connectivity
Waltmann M[3]	2019	20 healthy individuals with low and 19 with high positive schizotypy	Lower resting-state fMRI connectivity between ventromedial prefrontal regions and ventral striatal regions<br><br>Lower connectivity was also observed between the dorsal putamen and the hippocampus, occipital regions, as well as the cerebellum.
Derome M[4]	2018	75 healthy individuals. Examined anomalous self-experiences (ASE) on the mirror-gazing task	lower connectivity of specific visual areas within the primary visual network (PVN), and higher connectivity of regions within the Default Mode Network (DMN)

Wang Y[5]	2018	111 healthy individuals	Positively correlated with resting-state FC between the ventral striatum and frontal cortex<br><br>Negatively associated with resting-state FC between dorsal striatum and posterior cingulate.<br><br>Positive correlations with lateralization index of right dorsal caudate and right rostral putamen.
Zhu Y[6]	2017	20 individuals with schizotypal personality disorder and 19 healthy individuals	Decreased functional connectivity between the precuneus and contralateral parahippocampus
Wang Y[7]	2015	35 individuals with high schizotypy and 34 with low schizotypy	Reduced gray matter density in the insula and the dorsolateral prefrontal gyrus.<br><br>Weaker functional connectivity between the left insula and the putamen, but stronger connectivity between the cerebellum and the medial frontal gyrus.
Lagioia A[8]	2010	16 healthy individuals and 23 individuals seeking psychological help	(1) a default-mode network (DMN); (2) a dorsolateral attention network; (3) a visual network (VN); (4) an auditory network (AN); (5) a sensory motor network (SMN); (6) a self-referential network (SRN).

Supplementary Table 2: Summary of socio-demographic and clinical characteristics

**Attribute**	**Mean (SD)**	**Frequency**
**Age**	27.0 (5.2)	[illegible]
**Year of Education**	13.22 (3.73)	[illegible]
**Total SPQ**	4.42 (3.31)	[illegible]
**Total SIPS**	2.35 (6.41)	[illegible]
**Sex (M:F)**	[illegible]	42:15

**SPQ_01 (0:1)**	⊡	45:12
**SPQ_02 (0:1)**	⊡	55:2
**SPQ_03 (0:1)**	⊡	50:7
**SPQ_04 (0:1)**	⊡	49:8
**SPQ_05 (0:1)**	⊡	52:5
**SPQ_06 (0:1)**	⊡	53:4
**SPQ_07 (0:1)**	⊡	49:8
**SPQ_08 (0:1)**	⊡	50:7
**SPQ_09 (0:1)**	⊡	52:5
**SPQ_10 (0:1)**	⊡	51:6
**SPQ_11 (0:1)**	⊡	27:30
**SPQ_12 (0:1)**	⊡	43:13
**SPQ_13 (0:1)**	⊡	53:4
**SPQ_14 (0:1)**	⊡	29:28
**SPQ_15 (0:1)**	⊡	39:18
**SPQ_16 (0:1)**	⊡	46:11
**SPQ_17 (0:1)**	⊡	43:14
**SPQ_18 (0:1)**	⊡	48:9
**SPQ_19 (0:1)**	⊡	55:2
**SPQ_20 (0:1)**	⊡	49:8
**SPQ_21 (0:1)**	⊡	34:23
**SPQ_22 (0:1)**	⊡	30:27

Information on normative SPQ scores

In relation to the mean SPQ-B of schizophrenia positive group in comparison to a control population, we cite some earlier studies. Originally, Raine and Benishay (1995) presented normative data of 220 college students (from University of Southern California) as follows

Supplementary Table 3: Studies examining SPQ scores [Mean (SD)] in normative (control subjects) population

**Study**	**Cognitive-Perceptual**	**Interpersonal**	**Disorganized**	**Total**
**Raine *et al* 1995**[9]	3.6 (2.3)	3.6 (2.4)	2.5 (1.9)	9.6 (5.3)
**Compton *et al* 2007**[10]	1.8 (1.7)	2.7 (2.1)	1.1 (1.4)	5.6 (4.2)
**Solanki *et al*, 2012**[11]	0.8 (1.1)	1.5 (1.6)	0.8 (1.2)	3.2 (3.3)

We can see that these normative scores are higher than ones reported for subjects in our study, but this could be related to ethno-cultural differences. A newer study from the Southeastern United States (predominantly with African American population) showed the following profile of SPQ-B scores in non-psychiatric participants (n=57). Further, they showed 61 biological first-degree relatives of patients with schizophrenia also had similar mean total SPQ-B scores (5.0).

Control scores are variable between studies. Our data are derived from Indian population and our scores correspond well to other Indian population data. Solanki et al, 2012 reported normative values in the Indian subpopulation (smaller study, n = 30), which is where our sample was also collected.

Interestingly, Solanki et al, 2012 also examined the SPQ-B in first degree relatives of schizophrenia patients (n = 50), and found the mean total scores of 6.7 -- which closely matches the mean total score of schizophrenia-positive FDR subjects in our study. On other hand, the mean total score of our schizophrenia-negative FDR subjects closely matches that of their normative control group (3.2).

<u>**Supplementary References**</u>

1. Wang, Y. *et al.* Structural and functional brain abnormalities in children with schizotypal disorder: a pilot study. *NPJ Schizophr* **6**, 6 (2020).
2. Hua, J. P. Y. *et al.* Psychosis risk is associated with decreased resting-state functional connectivity between the striatum and the default mode network. *Cogn. Affect. Behav. Neurosci.* **19**, 998–1011 (2019).
3. Waltmann, M. *et al.* Multi-echo fMRI, resting-state connectivity, and high psychometric schizotypy. *Neuroimage Clin* **21**, 101603 (2019).
4. Derome, M. *et al.* Resting-State Networks of Adolescents Experiencing Depersonalization-Like Illusions: Cross-sectional and Longitudinal Findings. *Schizophr. Bull.* **44**, S501–S511 (2018).
5. Wang, Y., Ettinger, U., Meindl, T. & Chan, R. C. K. Association of schizotypy with striatocortical functional connectivity and its asymmetry in healthy adults. *Hum. Brain Mapp.* **39**, 288–299 (2018).
6. Zhu, Y. *et al.* Reduced functional connectivity between bilateral precuneus and contralateral parahippocampus in schizotypal personality disorder. *BMC Psychiatry* **17**, 48 (2017).
7. Wang, Y. *et al.* Neurobiological changes of schizotypy: evidence from both volume-based morphometric analysis and resting-state functional connectivity. *Schizophr. Bull.* **41 Suppl 2**, S444–54 (2015).
8. Lagioia, A., Van De Ville, D., Debbané, M., Lazeyras, F. & Eliez, S. Adolescent resting state networks and their associations with schizotypal trait expression. *Front. Syst. Neurosci.* **4**, (2010).
9. Raine, A. & Benishay, D. The SPQ-B: A Brief Screening Instrument for Schizotypal Personality Disorder. *Journal of Personality Disorders* vol. 9 346–355 (1995).

10. Compton, M. T., Chien, V. H. & Bollini, A. M. Psychometric properties of the Brief Version of the Schizotypal Personality Questionnaire in relatives of patients with schizophrenia-spectrum disorders and non-psychiatric controls. *Schizophr. Res.* **91**, 122–131 (2007).
11. Solanki, R. K., Swami, M. K., Singh, P. & Gupta, S. Identification of vulnerability among first-degree relatives of patients with schizophrenia. *East Asian Arch. Psychiatry* **22**, 118–125 (2012).
