# Supplementary material for: Extending schizophrenia diagnostic model to predict schizotypy in first-degree relatives
Source: NPJ Schizophr. 2020 Nov 6;6:30. doi: 10.1038/s41537-020-00119-y (PMC7648110; doi:10.1038/s41537-020-00119-y)
Supplement: Supplementary file 2 — Reporting Summary [file 41537_2020_119_MOESM2_ESM.pdf]

nature research

Corresponding author(s): Sunil Vasu Kalmady, G. Venkatasubramanian

Last updated by author(s): 2020-6-9

Reporting Summary

Nature Research wishes to improve the reproducibility of the work that we publish. This form provides structure for consistency and transparency in reporting. For further information on Nature Research policies, see our Editorial Policies and the Editorial Policy Checklist.

Statistics

For all statistical analyses, confirm that the following items are present in the figure legend, table legend, main text, or Methods section.

n/a	Confirmed	
☐	☒	The exact sample size (*n*) for each experimental group/condition, given as a discrete number and unit of measurement
☐	☒	A statement on whether measurements were taken from distinct samples or whether the same sample was measured repeatedly
☐	☒	The statistical test(s) used AND whether they are one- or two-sided<br>*Only common tests should be described solely by name; describe more complex techniques in the Methods section.*
☒	☐	A description of all covariates tested
☒	☐	A description of any assumptions or corrections, such as tests of normality and adjustment for multiple comparisons
☐	☒	A full description of the statistical parameters including central tendency (e.g. means) or other basic estimates (e.g. regression coefficient) AND variation (e.g. standard deviation) or associated estimates of uncertainty (e.g. confidence intervals)
☒	☐	For null hypothesis testing, the test statistic (e.g. *F*, *t*, *r*) with confidence intervals, effect sizes, degrees of freedom and *P* value noted<br>*Give P values as exact values whenever suitable.*
☒	☐	For Bayesian analysis, information on the choice of priors and Markov chain Monte Carlo settings
☒	☐	For hierarchical and complex designs, identification of the appropriate level for tests and full reporting of outcomes
☐	☒	Estimates of effect sizes (e.g. Cohen's *d*, Pearson's *r*), indicating how they were calculated

Our web collection on statistics for biologists contains articles on many of the points above.

Software and code

Policy information about availability of computer code

Data collection	R package 3, Microsoft Excel 2007
Data analysis	MATLAB R2016, SPM8, DPARSFA toolbox, Python 3, R package 3

For manuscripts utilizing custom algorithms or software that are central to the research but not yet described in published literature, software must be made available to editors and reviewers. We strongly encourage code deposition in a community repository (e.g. GitHub). See the Nature Research guidelines for submitting code & software for further information.

Data

Policy information about availability of data

All manuscripts must include a data availability statement. This statement should provide the following information, where applicable:

- Accession codes, unique identifiers, or web links for publicly available datasets
- A list of figures that have associated raw data
- A description of any restrictions on data availability

The datasets generated during and/or analysed during the current study are available from the corresponding author on a reasonable request.

Field-specific reporting

Please select the one below that is the best fit for your research. If you are not sure, read the appropriate sections before making your selection.

☐ Life sciences ☒ Behavioural & social sciences ☐ Ecological, evolutionary & environmental sciences

For a reference copy of the document with all sections, see nature.com/documents/nr-reporting-summary-flat.pdf

Life sciences study design

All studies must disclose on these points even when the disclosure is negative.

Sample size	*Describe how sample size was determined, detailing any statistical methods used to predetermine sample size OR if no sample-size calculation was performed, describe how sample sizes were chosen and provide a rationale for why these sample sizes are sufficient.*
Data exclusions	*Describe any data exclusions. If no data were excluded from the analyses, state so OR if data were excluded, describe the exclusions and the rationale behind them, indicating whether exclusion criteria were pre-established.*
Replication	*Describe the measures taken to verify the reproducibility of the experimental findings. If all attempts at replication were successful, confirm this OR if there are any findings that were not replicated or cannot be reproduced, note this and describe why.*
Randomization	*Describe how samples/organisms/participants were allocated into experimental groups. If allocation was not random, describe how covariates were controlled OR if this is not relevant to your study, explain why.*
Blinding	*Describe whether the investigators were blinded to group allocation during data collection and/or analysis. If blinding was not possible, describe why OR explain why blinding was not relevant to your study.*

Behavioural & social sciences study design

All studies must disclose on these points even when the disclosure is negative.

Study description	Quantitative Predictive Analysis
Research sample	This study examined 57 first-degree relatives of schizophrenia patients (M:F = 42:15) based on the following inclusion and exclusion criteria. We included siblings or children of schizophrenia patients, without any axis-1 disorder as evaluated by the Mini International Neuropsychiatric Interview (MINI) Plus. Probands of these participants were patients attending the clinical services of the National Institute of Mental Health & Neurosciences (NIMHANS), India, who fulfilled DSM-IV criteria for schizophrenia. The Structured Interview for Psychosis-risk Syndromes (SIPS) scale was administered to ascertain that these participants were unaffected by active psychosis. All except two subjects met criteria for 'Genetic Risk and Deterioration Prodromal Syndrome', while one subject met criteria for 'Attenuated Positive Symptom Prodromal Syndrome' and another for 'Brief Intermittent Psychotic Symptom Prodromal Syndrome'. We recruited only right-handed subjects to avoid potential confounds of differential handedness. No study subjects had contraindications to MRI or medical illness that could significantly influence brain structure/function, such as seizure disorder, cerebral palsy, or history suggestive of delayed developmental milestones. There was no history suggestive of DSM-IV psychoactive substance dependence or of head injury associated with loss of consciousness longer than 10 min. No participant had abnormal movements as assessed by the Abnormal Involuntary Movements Scale. Pregnant or postpartum females were not included. The age range was 17 to 38 years (27.2 ± 5.25 years). The catchment area for the subject recruitment involved the southern states of India. We obtained informed written consent after providing a complete description of the study to all the subjects. The NIMHANS ethics committee reviewed and approved the original research protocol. The Research Ethics Board at the University of Alberta, Edmonton approved the secondary analysis of archived data.
Sampling strategy	Convenience Sampling.<br>Sample size was adequate to produce stable predictions.
Data collection	We acquired and pre-processed a five-minute resting-state fMRI of each subject. A 22-item self-reported screening measure of schizotypal personality traits - Schizotypal Personality Questionnaire – Brief (SPQ-B) -- was used to assess the schizotypal personality score as an estimator of schizotypal expression for each participant (Range of total score: 0 to 22).
Timing	Start: October 2012<br>End: June 2016
Data exclusions	fMRI Images were checked for excessive head movement (translational > 2.0 mm and/or rotational > 2°). None were excluded.
Non-participation	Subjects who provided informed written consent participated in all of the data collection procedures. There were no dropouts since the study was a cross-sectional design.
Randomization	There were no experimental groups, and hence randomization was not required.

Ecological, evolutionary & environmental sciences study design

All studies must disclose on these points even when the disclosure is negative.

Study description	*Briefly describe the study. For quantitative data include treatment factors and interactions, design structure (e.g. factorial, nested, hierarchical), nature and number of experimental units and replicates.*
Research sample	*Describe the research sample (e.g. a group of tagged Passer domesticus, all Stenocereus thurberi within Organ Pipe Cactus National Monument), and provide a rationale for the sample choice. When relevant, describe the organism taxa, source, sex, age range and any manipulations. State what population the sample is meant to represent when applicable. For studies involving existing datasets, describe the data and its source.*
Sampling strategy	*Note the sampling procedure. Describe the statistical methods that were used to predetermine sample size OR if no sample-size calculation was performed, describe how sample sizes were chosen and provide a rationale for why these sample sizes are sufficient.*
Data collection	*Describe the data collection procedure, including who recorded the data and how.*
Timing and spatial scale	*Indicate the start and stop dates of data collection, noting the frequency and periodicity of sampling and providing a rationale for these choices. If there is a gap between collection periods, state the dates for each sample cohort. Specify the spatial scale from which the data are taken*
Data exclusions	*If no data were excluded from the analyses, state so OR if data were excluded, describe the exclusions and the rationale behind them, indicating whether exclusion criteria were pre-established.*
Reproducibility	*Describe the measures taken to verify the reproducibility of experimental findings. For each experiment, note whether any attempts to repeat the experiment failed OR state that all attempts to repeat the experiment were successful.*
Randomization	*Describe how samples/organisms/participants were allocated into groups. If allocation was not random, describe how covariates were controlled. If this is not relevant to your study, explain why.*
Blinding	*Describe the extent of blinding used during data acquisition and analysis. If blinding was not possible, describe why OR explain why blinding was not relevant to your study.*

Did the study involve field work? ☐ Yes ☐ No

Field work, collection and transport

Field conditions	*Describe the study conditions for field work, providing relevant parameters (e.g. temperature, rainfall).*
Location	*State the location of the sampling or experiment, providing relevant parameters (e.g. latitude and longitude, elevation, water depth).*
Access & import/export	*Describe the efforts you have made to access habitats and to collect and import/export your samples in a responsible manner and in compliance with local, national and international laws, noting any permits that were obtained (give the name of the issuing authority, the date of issue, and any identifying information).*
Disturbance	*Describe any disturbance caused by the study and how it was minimized.*

Reporting for specific materials, systems and methods

We require information from authors about some types of materials, experimental systems and methods used in many studies. Here, indicate whether each material, system or method listed is relevant to your study. If you are not sure if a list item applies to your research, read the appropriate section before selecting a response.

Materials & experimental systems

n/a	Involved in the study	
☒	☐	Antibodies
☒	☐	Eukaryotic cell lines
☒	☐	Palaeontology and archaeology
☒	☐	Animals and other organisms
☐	☒	Human research participants
☒	☐	Clinical data
☒	☐	Dual use research of concern

Methods

n/a	Involved in the study	
☒	☐	ChIP-seq
☒	☐	Flow cytometry
☐	☒	MRI-based neuroimaging

Antibodies

Antibodies used	*Describe all antibodies used in the study; as applicable, provide supplier name, catalog number, clone name, and lot number.*

Validation	*Describe the validation of each primary antibody for the species and application, noting any validation statements on the manufacturer's website, relevant citations, antibody profiles in online databases, or data provided in the manuscript.*

Eukaryotic cell lines

Policy information about cell lines

Cell line source(s)	*State the source of each cell line used.*
Authentication	*Describe the authentication procedures for each cell line used OR declare that none of the cell lines used were authenticated.*
Mycoplasma contamination	*Confirm that all cell lines tested negative for mycoplasma contamination OR describe the results of the testing for mycoplasma contamination OR declare that the cell lines were not tested for mycoplasma contamination.*
Commonly misidentified lines (See ICLAC register)	*Name any commonly misidentified cell lines used in the study and provide a rationale for their use.*

Palaeontology and Archaeology

Specimen provenance	*Provide provenance information for specimens and describe permits that were obtained for the work (including the name of the issuing authority, the date of issue, and any identifying information).*
Specimen deposition	*Indicate where the specimens have been deposited to permit free access by other researchers.*
Dating methods	*If new dates are provided, describe how they were obtained (e.g. collection, storage, sample pretreatment and measurement), where they were obtained (i.e. lab name), the calibration program and the protocol for quality assurance OR state that no new dates are provided.*

☐ Tick this box to confirm that the raw and calibrated dates are available in the paper or in Supplementary Information.

Ethics oversight	*Identify the organization(s) that approved or provided guidance on the study protocol, OR state that no ethical approval or guidance was required and explain why not.*

Note that full information on the approval of the study protocol must also be provided in the manuscript.

Animals and other organisms

Policy information about studies involving animals; ARRIVE guidelines recommended for reporting animal research

Laboratory animals	*For laboratory animals, report species, strain, sex and age OR state that the study did not involve laboratory animals.*
Wild animals	*Provide details on animals observed in or captured in the field; report species, sex and age where possible. Describe how animals were caught and transported and what happened to captive animals after the study (if killed, explain why and describe method; if released, say where and when) OR state that the study did not involve wild animals.*
Field-collected samples	*For laboratory work with field-collected samples, describe all relevant parameters such as housing, maintenance, temperature, photoperiod and end-of-experiment protocol OR state that the study did not involve samples collected from the field.*
Ethics oversight	*Identify the organization(s) that approved or provided guidance on the study protocol, OR state that no ethical approval or guidance was required and explain why not.*

Note that full information on the approval of the study protocol must also be provided in the manuscript.

Human research participants

Policy information about studies involving human research participants

Population characteristics	See above
Recruitment	See above
Ethics oversight	The National Institute of Mental Health & Neurosciences (NIMHANS), India ethics committee reviewed and approved the original research protocol. The Research Ethics Board at the University of Alberta, Edmonton approved the secondary analysis of archived data.

Note that full information on the approval of the study protocol must also be provided in the manuscript.

Clinical data

Policy information about clinical studies

All manuscripts should comply with the ICMJE guidelines for publication of clinical research and a completed CONSORT checklist must be included with all submissions.

Clinical trial registration	*Provide the trial registration number from ClinicalTrials.gov or an equivalent agency.*

Study protocol | *Note where the full trial protocol can be accessed OR if not available, explain why.*

Data collection | *Describe the settings and locales of data collection, noting the time periods of recruitment and data collection.*

Outcomes | *Describe how you pre-defined primary and secondary outcome measures and how you assessed these measures.*

Dual use research of concern

Policy information about dual use research of concern

Hazards

Could the accidental, deliberate or reckless misuse of agents or technologies generated in the work, or the application of information presented in the manuscript, pose a threat to:

No	Yes	
☐	☐	Public health
☐	☐	National security
☐	☐	Crops and/or livestock
☐	☐	Ecosystems
☐	☐	Any other significant area

Experiments of concern

Does the work involve any of these experiments of concern:

No	Yes	
☐	☐	Demonstrate how to render a vaccine ineffective
☐	☐	Confer resistance to therapeutically useful antibiotics or antiviral agents
☐	☐	Enhance the virulence of a pathogen or render a nonpathogen virulent
☐	☐	Increase transmissibility of a pathogen
☐	☐	Alter the host range of a pathogen
☐	☐	Enable evasion of diagnostic/detection modalities
☐	☐	Enable the weaponization of a biological agent or toxin
☐	☐	Any other potentially harmful combination of experiments and agents

ChIP-seq

Data deposition

☐ Confirm that both raw and final processed data have been deposited in a public database such as GEO.

☐ Confirm that you have deposited or provided access to graph files (e.g. BED files) for the called peaks.

Data access links
May remain private before publication. | *For "Initial submission" or "Revised version" documents, provide reviewer access links. For your "Final submission" document, provide a link to the deposited data.*

Files in database submission | *Provide a list of all files available in the database submission.*

Genome browser session
(e.g. UCSC) | *Provide a link to an anonymized genome browser session for "Initial submission" and "Revised version" documents only, to enable peer review. Write "no longer applicable" for "Final submission" documents.*

Methodology

Replicates | *Describe the experimental replicates, specifying number, type and replicate agreement.*

Sequencing depth | *Describe the sequencing depth for each experiment, providing the total number of reads, uniquely mapped reads, length of reads and whether they were paired- or single-end.*

Antibodies | *Describe the antibodies used for the ChIP-seq experiments; as applicable, provide supplier name, catalog number, clone name, and lot number.*

Peak calling parameters | *Specify the command line program and parameters used for read mapping and peak calling, including the ChIP, control and index files used.*

Data quality | *Describe the methods used to ensure data quality in full detail, including how many peaks are at FDR 5% and above 5-fold enrichment.*

Software | *Describe the software used to collect and analyze the ChIP-seq data. For custom code that has been deposited into a community repository, provide accession details.*

Flow Cytometry

Plots

Confirm that:

☐ The axis labels state the marker and fluorochrome used (e.g. CD4-FITC).

☐ The axis scales are clearly visible. Include numbers along axes only for bottom left plot of group (a 'group' is an analysis of identical markers).

☐ All plots are contour plots with outliers or pseudocolor plots.

☐ A numerical value for number of cells or percentage (with statistics) is provided.

Methodology

Sample preparation	*Describe the sample preparation, detailing the biological source of the cells and any tissue processing steps used.*
Instrument	*Identify the instrument used for data collection, specifying make and model number.*
Software	*Describe the software used to collect and analyze the flow cytometry data. For custom code that has been deposited into a community repository, provide accession details.*
Cell population abundance	*Describe the abundance of the relevant cell populations within post-sort fractions, providing details on the purity of the samples and how it was determined.*
Gating strategy	*Describe the gating strategy used for all relevant experiments, specifying the preliminary FSC/SSC gates of the starting cell population, indicating where boundaries between "positive" and "negative" staining cell populations are defined.*

☐ Tick this box to confirm that a figure exemplifying the gating strategy is provided in the Supplementary Information.

Magnetic resonance imaging

Experimental design

Design type	Resting State fMRI
Design specifications	Scan duration of 5 minutes 14 seconds, yielding 153 dynamic scans.
Behavioral performance measures	Subjects were asked to keep their eyes open during the scan.

Acquisition

Imaging type(s)	Functional, Structural (used only for Co-registration)
Field strength	3.0 Tesla
Sequence & imaging parameters	Magnetic Resonance Imaging (MRI) was done in a 3.0 Tesla scanner (Magnetom Skyra, Siemens). Resting State Functional MRI: BOLD (Blood Oxygen Level Dependent) sensitive echo-planar imaging was obtained using a 32-channel coil for a duration of 5 minutes 14 seconds, yielding 153 dynamic scans. The scan parameters were: TR= 2000 msec; TE = 30 msec; flip angle = 78 degrees; Slice thickness = 3 mm; Slice order: Descending; Slice number = 37; Gap = 25%; Matrix = 64 × 64 × 64 mm3, FOV = 192×192, voxel size = 3.0 mm isotropic. Subjects were asked to keep their eyes open during the scan. For intra-subject co-registration, structural MRI: T1 weighted three-dimensional high-resolution MRI was performed (TR = 8.1 msec, TE = 3.7 msec, nutation angle = 8 degree, FOV = 256 mm, slice thickness = 1 mm without inter-slice gap, NEX = 1, matrix = 256×256) yielding 165 sagittal slices.
Area of acquisition	Whole Brain

Diffusion MRI ☐ Used ☒ Not used

Preprocessing

Preprocessing software | We visually inspected the acquired images for artifacts such as incomplete brain coverage or ghosting; then re-orientated the origin to the anterior commissure in structural MRI and fMRI images. Then, for each subject, we discarded the first ten volumes of each functional time-series before reaching steady magnetization and for allowing participants to adapt to scanning noise. Images were then pre-processed with slice-timing correction and image realignment to correct for motion. Functional images were co-registered with the structural image and then normalized to MNI space resampled to 3 × 3 × 3 mm3. Further, we performed nuisance regression to denoise signal induced by head motion using 24 regressors derived from the parameters estimated during motion realignment, scanner drift using a linear term, as well as global fMRI signals from white matter and cerebrospinal fluid segments using SPM's new segment method. Finally, we smoothed, detrended and band-pass filtered (0.01–0.08 Hz) the normalized images. Software packages used for pre-processing and feature extraction [illegible]

are Statistical parametric mapping (SPM8, http://www.fil.ion.ucl.ac.uk/spm), Data Processing Assistant for Resting-State fMRI (DPARSF), and nilearn python package.

Normalization	Functional images were co-registered with the structural image and then normalized to MNI space resampled to 3 × 3 × 3 mm3.
Normalization template	MNI152
Noise and artifact removal	a) We checked acquired images visually for artefacts such as incomplete brain coverage or ghosting.<br>b) fMRI Images were checked for excessive head movement (translational > 2.0 mm and/or rotational > 2°). None were excluded.<br>c) Nuisance regression was performed to remove noise in the signal induced by head motion using 24 regressors derived from the parameters estimated during motion realignment, scanner drift using a linear term, as well as global fMRI signals from white matter and cerebrospinal fluid segments using SPM's new segment method.
Volume censoring	Not performed

Statistical modeling & inference

Model type and settings	Predictive (Machine learning) analysis.
Effect(s) tested	This study examines whether a schizophrenia diagnosis model, learned using schizophrenia and normal fMRI datasets, can identify higher schizotypal scores in first degree relatives without schizophrenia.

Specify type of analysis: ☐ Whole brain ☐ ROI-based ☒ Both

Statistic type for inference (See Eklund et al. 2016)	No standard statistical inferences were made on neuroimaging data. Instead, the study focuses on prediction accuracy.
Correction	No standard statistical inferences were made on neuroimaging data. Instead, the study focuses on prediction accuracy.

Models & analysis

n/a	Involved in the study
☐	☐ Functional and/or effective connectivity
☐	☐ Graph analysis
☐	☒ Multivariate modeling or predictive analysis

Functional and/or effective connectivity	*Report the measures of dependence used and the model details (e.g. Pearson correlation, partial correlation, mutual information).*
Graph analysis	*Report the dependent variable and connectivity measure, specifying weighted graph or binarized graph, subject- or group-level, and the global and/or node summaries used (e.g. clustering coefficient, efficiency, etc.).*
Multivariate modeling and predictive analysis	Recently, we developed a machine-learning algorithm 'EMPaSchiz' that learns, from a training set of schizophrenia patients and healthy individuals, a model that predicts if a novel individual has schizophrenia, based on features extracted from his/her resting-state functional MRI (fMRI).<br><br>Kalmady, S. V. et al. Towards artificial intelligence in mental health by improving schizophrenia prediction with multiple brain parcellation ensemble-learning. NPJ Schizophr 5, 2 (2019).<br><br>In this study, we apply this learned model to first degree relatives of schizophrenia patients, who were found to not have active psychosis or schizophrenia. The study examines whether a schizophrenia diagnosis model, learned using schizophrenia and normal fMRI datasets, can identify higher schizotypal scores in first degree relatives without schizophrenia.
